# Fast learning in free-foraging bumble bees is negatively correlated with lifetime resource collection

**DOI:** 10.1038/s41598-017-00389-0

**Published:** 2017-03-29

**Authors:** Lisa J. Evans, Karen E. Smith, Nigel E. Raine

**Affiliations:** 1 School of Biological Sciences, Royal Holloway University of London, Egham, Surrey TW20 0EX UK; 2 The New Zealand Institute for Plant and Food Research, Hamilton, 3240 New Zealand; 3 School of Environmental Sciences, University of Guelph, Guelph, Ontario N1G 2W1 Canada

## Abstract

Despite widespread interest in the potential adaptive value of individual differences in cognition, few studies have attempted to address the question of how variation in learning and memory impacts their performance in natural environments. Using a novel split-colony experimental design we evaluated visual learning performance of foraging naïve bumble bees (*Bombus terrestris*) in an ecologically relevant associative learning task under controlled laboratory conditions, before monitoring the lifetime foraging performance of the same individual bees in the field. We found appreciable variation among the 85 workers tested in both their learning and foraging performance, which was not predicted by colony membership. However, rather than finding that foragers benefited from enhanced learning performance, we found that fast and slow learners collected food at comparable rates and completed a similar number of foraging bouts per day in the field. Furthermore, bees with better learning abilities foraged for fewer days; suggesting a cost of enhanced learning performance in the wild. As a result, slower learning individuals collected more resources for their colony over the course of their foraging career. These results demonstrate that enhanced cognitive traits are not necessarily beneficial to the foraging performance of individuals or colonies in all environments.

## Introduction

The question of why cognitive abilities, such as learning and memory, vary so widely within species is one of the most intriguing, yet unanswered, issues surrounding the evolution of cognitive traits^1–5^. It is commonly assumed that differences in cognitive ability directly affect fitness^6^, and whilst studies conducted under controlled laboratory conditions provide some evidence supporting this view^7–10^, it is notoriously difficult to assess what impact variation in cognitive ability has on animal performance in the wild^5, 11, 12^. One reason for this is the difficulty of designing a task simple enough to be used by individuals in the wild, yet sufficiently robust to reveal variation in a focal cognitive trait. For instance, several groups have conducted experiments to assess individual problem-solving ability (e.g. male satin bowerbirds required to remove red objects from their bowers^13^) as a means of testing cognitive complexity/intelligence^2, 14–17^. However, such problem-solving tasks may actually be measuring variation in factors such as neophobia, motivation, persistence or strength rather than the cognitive traits of interest. Interpreting variation in cognitive ability among individuals in wild populations e.g. refs 2, 13, 14, 16–18 is also challenging because it is impossible to know the experience of these individuals prior to capture/testing or, more importantly, how such experience might differ among individuals.

To reliably generate standardized measures of variation in cognitive traits, naïve individuals must be subjected to laboratory assessments that are removed from social cues and environmental variables, thus minimising variation in other traits or stochastic effects. However, this approach also has potential limitations as the lack of natural setting and social context in a laboratory environment could mean that subject choices will not reflect the choices those animals would make in the wild. For example, Morand-Ferron and Quinn^15^ found that individual great tits (*Parus major*) and blue tits (*Cyanistes caeruleus*) displayed more frequent and efficient problem-solving in larger groups than when tested on their own or in small groups. Thus forming an association between laboratory-based behavioural assays and field studies is critical if we are to understand how cognitive abilities (such as learning and memory performance) truly impact performance and fitness^2, 19^.

Bumble bees provide a tractable study system that can be used to address these questions. Colonies can be purchased at an early developmental stage (or raised from wild-caught queens) allowing experimenters to control for differences in experience (as well as the size and age) of tested individuals. Colonies are also amenable to laboratory conditions enabling us to assess individual learning ability, a distinct cognitive trait, from successive trials. In the field, colony foraging performance provides a robust proxy measure of colony fitness as it correlates well with production of sexual offspring: new queens (gynes) and males^20–22^. Whilst quantifying the behaviour of individual bumble bees offers many practical advantages, they are social insects, so natural selection also acts on the behaviour of the colony as a whole, rather than just the behaviour of each individual. However, recent studies have demonstrated that there can be a direct functional relationship between both individual behavioural types and colony-level behaviour (e.g. boldness and foraging aggressiveness in social spiders *Stegodyphus sarasinorum* and *S. dumicola* ^23–26^), and also between the performance of individual workers and the success of the entire colony (e.g. aggressiveness and number of offspring colonies produced by the social spider *Anelosimus studiosus* and foraging performance and colony growth in bumble bees *Bombus terrestris* ^27–29^). Therefore, by quantifying the behaviour/performance of individuals within a colony we can make strong predictions about colony-level performance.

Here we assess whether individual cognitive performance, specifically learning ability, of naïve bumble bee (*B. terrestris*) workers is correlated with their lifetime foraging performance/contribution to their colony: i.e. do the fastest learners collect more of their colony’s food resources? Learning is of critical importance to flower-visiting insects because they must learn which flowers provide rewards, when these blooms are most productive, where to find rewarding flowers, and how to extract this nectar and pollen^30^. We hypothesize that fast learning individuals will be better able to respond to these changing demands and therefore collect more food from flowers during their field foraging career.

To test our hypothesis we measured individual learning performance using an ecologically realistic visual (colour/reward association) learning task in the laboratory. The foraging activity of the same individuals was subsequently monitored in the field using Radio Frequency Identification (RFID) tagging technology. We routinely monitored the quantity of nectar or pollen brought back to the nest and recorded the duration of all foraging trips undertaken by our tested individuals.

## Results

### Individual learning performance

**Figure 1:**
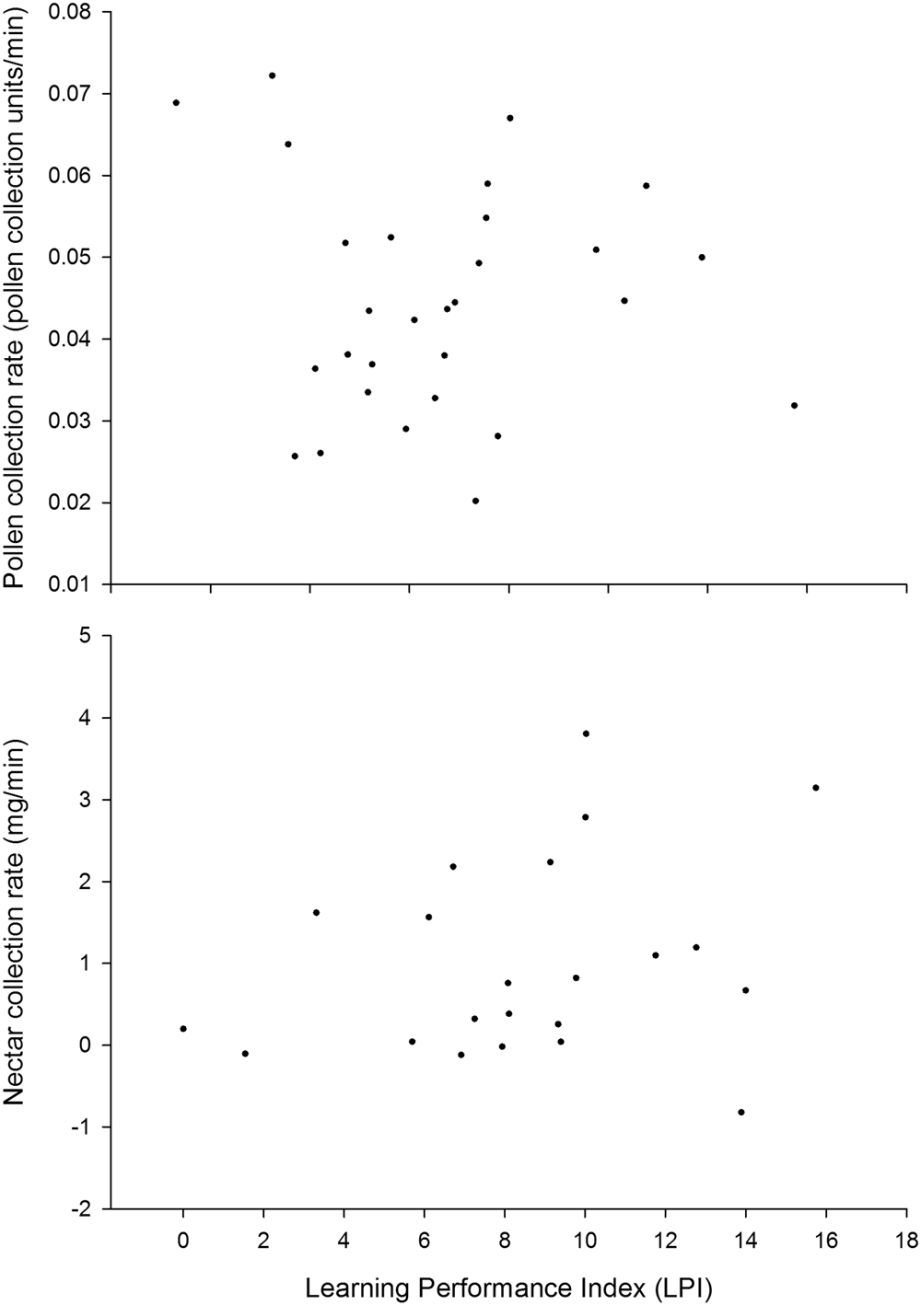
Correlations between (**a**) mean pollen collection rates (pollen score/min) and LPI of 30 bees that were observed to perform at least three pollen foraging bouts and (**b**) mean nectar collection rates (mg/min) and Learning Performance Index (LPI) of the 22 bees that were observed to perform at least three nectar foraging bouts. Lower LPI values indicate that the bee was a faster learner (i.e. made fewer errors). Neither correlation is statistically significant.

How does visual learning performance vary among individuals? We compared the visual learning performance (see methods) of 85 bees across five colonies (n = 15–21 bees per colony). Whilst we found marked differences in the learning performance index (LPI) of individual workers (e.g. Fig. 1a), that varied between 0.1 (fast learner) and 15.8 (slow learner), there was no significant difference in LPI among the five natal colonies (one-way ANOVA F_(4,81)_ = 1.51, P = 0.21). Furthermore, LPI was not correlated with any of the variables that were identified as potential covariates, including the age of the foragers when their learning performance was assessed (Spearman’s ρ = −0.027, n = 49, P = 0.06), the age of the colony when learning performance of each forager was assessed (Spearman’s ρ = −0.22, n = 49, P = 0.13), or forager mass (Spearman’s ρ = −0.02, n = 49, P = 0.91).

### Individual foraging efficiency

Does learning performance predict nectar and/or pollen collection rates? All 85 bees whose learning performance was assessed were RFID tagged and allowed to forage in the natural environment surrounding the laboratory (Egham, Surrey, UK); of these 58% (n = 49/85) were classified as foragers as they completed more than five foraging bouts; the other 36 (42%) tested bees did not forage for their colony (all but one bee left the colony, and did not return, without completing a single foraging bout). We found no evidence that the likelihood of bees returning to their colony was related to individual learning performance (Mann-Whitney U: z = 1.22, P = 0.22).

The RFID tagging technology automatically recorded when each bee left and re-entered the nest, yielding data from 4619 foraging bouts (made by the 49 foragers), with each bee completing between 6–253 bouts (mean ± SE = 103 ± 9). Each colony was also monitored by two observers for a total of 60 hours to determine the amount of pollen and nectar collected by foraging bees. Foraging efficiency (the amount of pollen or nectar collected by each forager per unit of time outside the nest) was observed for 9.4% of the foraging bouts undertaken by 92% (n = 45/49 bees) of the tagged foragers. The number of bouts for which foraging efficiency was observed was directly proportional to the total number of foraging bouts undertaken (recorded using the RFID system) by each bee (Spearman’s ρ = 0.76, n = 45, P < 0.001).

Comparing candidate models (that contained colony membership as a random effect and one of the following covariates as a fixed effect: colony age, worker age, worker mass or experience (See Section S4, for definitions) and then adding learning performance to the best model) we found that learning performance did not predict either pollen or nectar collection rate, indicating that there was no predictable difference in the foraging efficiency of bees with strong or poor learning ability (Table 1; Fig. 1a,b). Experience was a good predictor of nectar collection rate: estimate ± SE = 0.01 ± 0.003. Bees that had higher experience scores (completed more nectar foraging trips) collected nectar at a higher rate.

**Table 1:** Candidate models to predict pollen and nectar collection efficiency by tested bees.

	Mean pollen collection		Mean nectar collection	
	AICc	Δ AICc	AICc	Δ AICc
Basic	−164.33*	**0**	76.77	14.46
Worker age	−162.59	1.74	78.13	15.82
Worker size	−161.82	2.51	79.71	17.41
Colony age	−161.96	2.36	65.90	3.60
Experience	−162.54	1.79	62.31*	**0**
Best model + LPI	−163.33	1.00	65.70	3.39

The basic model contained only the intercept and colony membership as a random factor. All other models contained the basic model and the additional fixed factors (predictors) specified in the model name. The model with the lowest AICc value out of the five initial models (indicated with an asterisk) had learning ability performance (LPI) added to it to determine whether this significantly decreased the AICc value (i.e. Δ AICc >2). The best model (based on the AICc value) is shown in bold.

### Individual foraging activity

**Figure 2:**
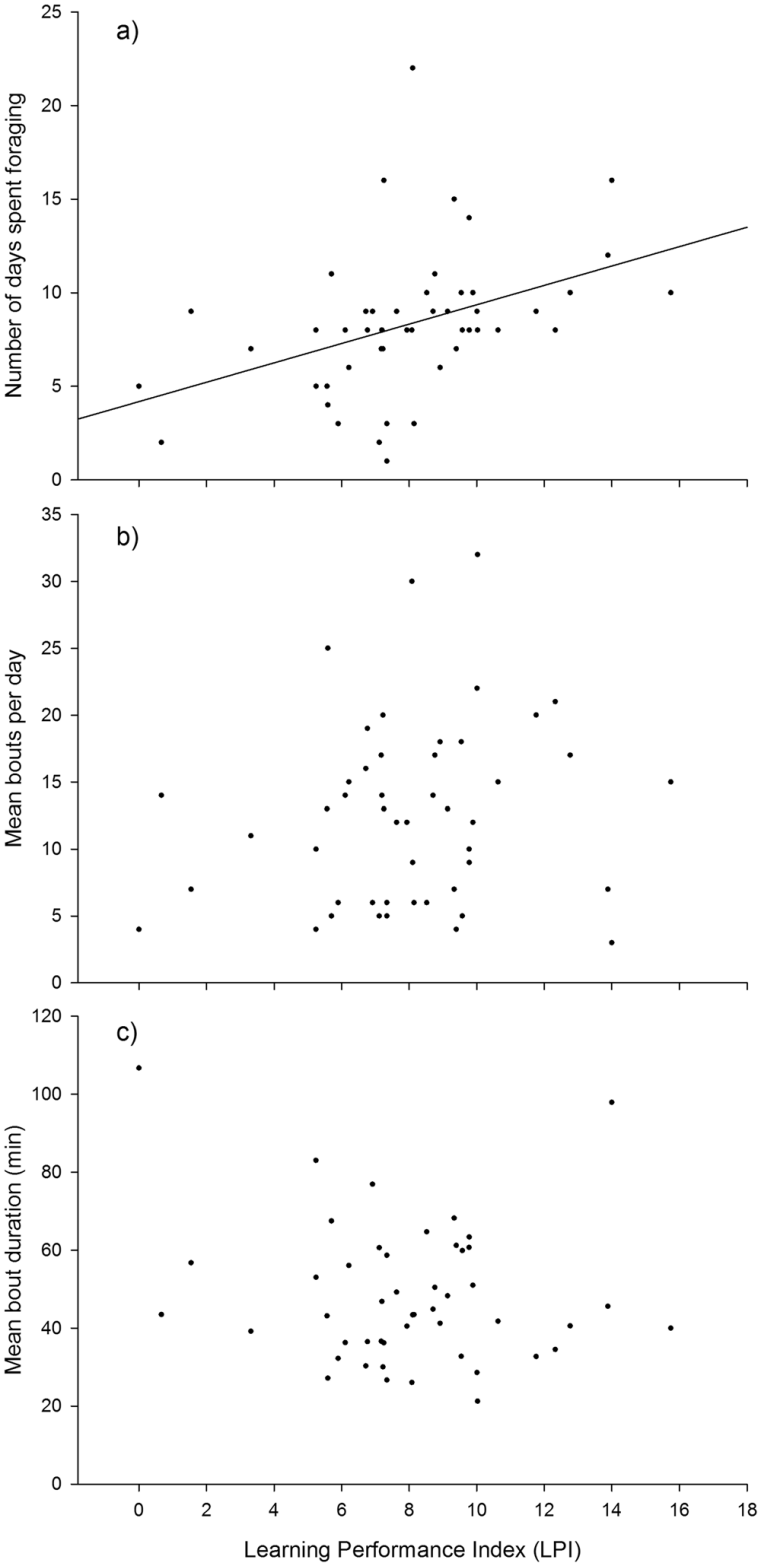
Correlations between Learning Performance Index (LPI) and (**a)** the number of days on which each bee foraged, (**b**) the mean number of foraging bouts undertaken per day and (**c**) the mean duration of foraging bouts. A line of best fit, generated from a least-square linear regression, has been added for ease of interpretation to the significant correlation in panel a. Data presented are for all 49 bees that were classified as ‘foraging’ once they were RFID tagged, with each dot representing a single bee. Figures do not describe ‘colony membership’ as this was included as a random factor in the best fitting model. Lower LPI values indicate that the bee was a faster learner (i.e. made fewer errors).

Does learning performance predict the amount of foraging undertaken by bees? Assessed bees (n = 49) foraged for 1–22 days (Fig. 2a; mean ± SE = 8.33 ± 0.55), completing 3–36 foraging bouts per day (Fig. 2b; mean ± SE = 12.50 ± 0.56), with each bout lasting 21–106 minutes (Fig. 2c; mean ± SE = 48.25 ± 2.55). Comparing candidate models we found that the number of days spent foraging was best predicted by a model containing learning performance (LPI: estimate ± SE = 0.06 ± 0.02; Table 2). LPI was significantly positively correlated with number of days spent foraging (Fig. 2a). As faster learning bees (i.e. those that made fewer errors) had lower LPI values, our results indicate that faster learners foraged for fewer days. The number of days on which bees foraged appeared to be a good proxy measure of individual foraging lifespan, because once a bee began foraging in the field they almost all (92%) continued foraging for consecutive days until the end of their life. We assume foraging continued until death because the period for which they were away from the colony increased as they approached their final foraging bout (Fig. S5), a likely consequence of senescence^31^.

**Table 2:** Candidate models to predict the number of days foraged, the mean number of bouts per day and mean bout duration.

	No. of days foraged		Mean bouts per day		Mean bout duration	
	AICc	Δ AICc	AICc	Δ AICc	AICc	Δ AICc
Basic	90.59*	12.35	326.25	25.54	161.48	7.80
Worker age	91.14	12.89	322.75	22.05	155.86	2.17
Worker size	93.36	15.12	326.37	25.66	163.53	9.84
Colony age	92.54	14.30	300.71*	**0**	153.69*	**0**
Best model + LPI	**78.24**	**0**	303.16	2.46	156.04	2.35

The basic model contained only the intercept and colony membership as a random factor. All other models contained the basic model and the additional fixed factors (predictors) specified in the model name. The model with the lowest AICc value out of the five initial models (indicated with an asterisk) had learning performance (LPI) added to it to determine whether this significantly decreased the AICc value (i.e. Δ AICc >2). The best model (based on the AICc value) is shown in bold.

Learning performance did not predict either the mean number of foraging bouts a bee undertook per day or mean bout duration (Table 2; Fig. 2b,c), indicating that fast and slow learners completed a similar number of bouts each day, that were similar in duration (in both cases, colony age was the best predictor: estimate ± SE = −0.52 ± 0.085 and 0.06 ± 0.02 respectively). Bees transferred to the external side of the nest (so they could forage in field conditions) when colonies were older completed fewer foraging bouts per day; this may reflect a reduced demand for food resources as worker production began to decrease. These bees also spent more time away from the colony on each trip. This increase in bout duration may reflect a reduction in food sources after six weeks of hot/dry weather during the experimental period (Fig. S6).

## Discussion

To determine whether the cognitive abilities of individual bumble bees predict foraging performance in their natural environment, we assessed learning performance using a colour/reward association task in the laboratory, then monitored their foraging activity in the field. We found that learning performance did not predict nectar or pollen collection rates; bees collected these resources at similar rates from real flowers in the field irrespective of their performance in the laboratory learning task. However, learning performance did predict foraging activity level. Slower learners foraged for more days whilst still completing a similar number of bouts per day. Consequently slower learners completed a greater number of foraging bouts overall.

Our results suggest that slower learners made a greater contribution to their colony’s food resources because these individuals foraged for more days. This result is surprising because enhanced learning performance is typically assumed to be beneficial: a view consistent with results from Raine and Chittka^19^ who showed that colonies containing slower (on average) visual learners brought back 40% less nectar than faster learning colonies. Importantly, the current study explicitly followed the foraging performance of workers for which learning had previously been assessed while Raine and Chittka^19^ used different individuals. These authors removed tested individuals (in order to recruit new foragers when assessing their learning performance) before field observations. Thus they relied on the assumption that the learning performance of the tested workers reflected that of workers in the field. Previous research has demonstrated the learning performance of individual bumble bees within a colony can vary significantly depending upon colony developmental stage, and worker reproductive status, at the time the assessments take place^32, 33^. Consequently the mean learning performance of the laboratory-tested workers in Raine and Chittka’s^19^ study may not be the best representation of colony learning performance when transferred into the field.

Our approach of using the same individuals in the lab and field may have meant that our associative learning task interfered with subsequent foraging behaviour. For instance, it is possible that bees learning the yellow/reward association quickly found it more difficult to adapt to the new outdoor foraging task. However, faster learners have been previously shown to reverse learned associations more quickly^34^, suggesting that they will readily learn to forage on a different flower type if the previous flower type is no longer present or rewarding.

Raine and Chittka’s^19^ study, which used the same associative learning task, may have also produced contrasting results to this study because of differences in resource availability and distribution in the test landscape (urban vs. rural/residential), number of colonies tested, presence or absence of supplemental pollen feeding, and/or potential stochastic variation in colony-level variables (e.g. food stores, queen condition or parasite load). Most of these factors can be determined to some extent by experimenters through colony placement and/or empirical design. In our current study we mitigate the hard-to-avoid caveat of potentially confounding additional colony-level variables by determining as much as possible about the life histories of all the individual workers tested and controlling for any important differences statistically.

One hypothesis to explain why faster learners foraged for fewer days is that the costs associated with enhanced cognitive performance reduced their foraging lifespan^35–39^. Comparative differences in learning performance among bumble bees have been shown to be consistent over time (three-four weeks)^40^, thus foragers that were fast learners when tested in the lab are likely to have remained fast learners once out in the field. Neural tissue is metabolically expensive to produce and maintain, and the storage of new and the removal of old information is likely to require energy and other resources^41^. By investing energy in producing and maintaining the neural tissue used in learning and memory, these individuals may be trading this off against other traits, such as the length of their foraging career. Whilst the current study was not designed to measure foraging lifespan, the number of days these bees foraged for was almost always consecutive (92% of bees) and they continued foraging until their death. Thus the number of days spent foraging provides an indication of foraging career duration/foraging lifespan, in which case, our fastest learning individuals had significantly shorter foraging lifespans. An ‘energetic cost’ associated with learning would be consistent with results from honey bees (*Apis mellifera*), for which a negative correlation has been shown between learning performance and survival (when food was withheld) under laboratory conditions^38^.

Whilst an energetic cost associated with learning could explain why faster learners foraged for fewer days (completing fewer foraging bouts overall), it does not explain why they did not collect nectar and/or pollen at a higher rate. It is generally thought that the ability to learn salient floral cues, such as colour, rapidly would assist foragers to track changes in rewards that are highly variable among plant species and change over time^40, 42^. In a recent review, Rowe and Healy^12^ question whether it is realistic to expect cognition to be correlated with fitness. These authors argue that we should not expect to see ‘smart’ individuals being selected for, but rather individuals that are ‘smart’ for their environment, i.e. their cognitive abilities are optimized for their particular environment. Previous authors also suggest that learning is only valuable to an animal in a context-dependent fashion, i.e. in some circumstances learning information will be vital but at other times costly^3, 43^. Thus the ability for flower visiting insects to learn which are the best flowers to visit is likely to be more important in some environments than others. For example, the honey bee waggle dance (a sophisticated communication system reliant on social learning, which is used to locate food) was only found to be beneficial for foraging in habitats such as tropical forests, where food sources are harder to find, variable in reward quantity and ephemeral. In other environments (such as temperate habitats), in which floral resources were more abundant and widespread, experimental interference with dance behaviour did not affect resource collection success^44, 45^ (but see ref. 46). Results from our study suggest that in the rural/residential landscape surrounding the test site at Royal Holloway, the ability to learn quickly did not confer an advantage in terms of foraging efficiency. However, that does not mean that enhanced learning would not be beneficial under a different set of environmental conditions, e.g. a city centre landscape as used by^19^.

There may also be more than one way to be a ‘smart’ bumble bee forager. Slower learning bees could acquire and use information in different ways, possibly employing alternative foraging strategies. We know that in some environments alternative approaches allow bees to perform comparably or even better than fast learners^47–49^. For instance, when foraging in a patch containing two similarly coloured artificial flowers, one colour containing sucrose solution the other unrewarding, fast, inaccurate honey bees (slow learners) had a higher nectar collection rate when many of the flowers contained rewards^47, 48^. Furthermore, bumble bees that make more visits to unrewarded flowers (foraging errors) are more likely to sample alternative flower species. Such exploration can lead them to discover more highly rewarding flowers that in turn can increase their foraging performance^49^. Taken together, these findings suggest that in some environments the foraging efficiency of slower learners could be comparable to faster learners.

It should be noted that we measured a single form of learning, visual (colour) learning. When locating and extracting rewards from floral resources, foraging bees are likely to use cues from multiple sensory modalities (e.g. olfactory, visual and tactile) and employ other forms of learning (e.g. spatial and flower handling). The relative importance of these different sensory modalities and forms of learning is not well understood, but to some extent their importance is likely to depend on context and/or environment. For instance, visual cues will be less useful under low light conditions^50^. There is some evidence to suggest that individuals that learn rapidly in one modality will not necessarily perform similarly well in another modality^51^, (but see ref. 52). Therefore, it would be interesting to measure learning using different modalities and tasks and see how these are related to foraging performance.

In conclusion, rather than finding a selective advantage for faster learning, our results suggest that enhanced learning ability does not predict foraging efficiency and may come at an energetic cost. As such, this study provides the first evidence of a learning associated cost in the wild. However, because faster learning appears to be associated with greater cost to the individual (and perhaps also the colony), as these bees had a shortened foraging career, it seems unlikely that such a trait would be maintained within the population without a selective advantage. As with other behaviours there are potential benefits for this cognitive variation within bumble bee colonies: it could promote efficient patterns of task allocation (division of labour)^53–56^ and/or increase colony flexibility/resilience when faced with external disturbance or change in their environment^57–60^ (but see refs 61, 62). To determine whether there is a ‘particular’ environment that favours enhanced learning, future studies measuring cognitive traits and individual performance should consider assessing learning across multiple tasks and also using a variety of environments.

## Methods

Five colonies, containing between 25–38 workers (mean = 30), were obtained from Biobest (Westerlo, Belgium). The bees and brood in each colony were divided equally between two brood chambers of a colony box divided by a plastic mesh (mesh size: 1 × 1 mm), allowing bees on either side to maintain colony cohesion through odour (pheromonal) and tactile communication (Fig. S1). The queen was switched between the sides every 24 hours to prevent the colony from perceiving it was queenless^20^. This divided colony box setup enabled us to connect one side (internal) to a flight arena, in which foragers without prior foraging experience were assessed in a visual learning paradigm (described below). The other side (external) of the colony box was connected to the window (Figs S2 and 3), providing foragers access to the natural environment surrounding the laboratory. This enabled us to monitor the free-foraging performance of our tested individuals in the field.

All newly emerged workers on the internal side of each colony were individually marked with numbered tags (Opalith tags; Christian Graze KG, Germany) on the day of emergence, so their age when tested was known. On completion of the learning task, foragers were re-tagged with an RFID tag on top of their Opalith tag Microsensys GmbH: mic3-Tag 64 bit read only transponder; carrier frequency: 13.56 MHz; measuring: 2 × 1.6 × 0.5 mm; mass: 4 mg – which does not affect a bee’s behaviour^29, 63–65^. The identification number of the RFID tag was recorded along with the bee’s Opalith tag number. Each bee was then transferred into the external side of its corresponding colony so its foraging behaviour could be monitored in the field. To ensure that all colony foraging was performed by tested bees transferred from the internal side, workers emerging in the external side had one of their wings clipped to prevent them flying and foraging.

No external sides were connected to the outside until the learning performance of at least two foraging workers had been tested (7–14 days after colonies arrived). In this initial period, each side of the colony was provided with 3 g of defrosted honey bee-collected pollen (sourced from Koppert Ltd UK) every second day, and *ad libitum* (50% v/v) sucrose solution in a colourless, transparent feeder. Once the external side had been connected to the outside, each colony was given no additional pollen and only sufficient sucrose solution to fill three nectar pots. The internal side of each colony was given access to *ad libitum* sucrose (50% v/v) in the flight arena except when the learning task was being assessed, and 3 g of pollen was provided directly into the brood chamber every second day.

### Forager learning performance

The ability of bees (n = 85 across the 5 colonies, range = 15–21) to learn to associate the colour yellow with a sucrose reward was assessed using an established visual learning task^19, 66^. Individual workers foraged in an array of 10 blue (empty) and 10 yellow (containing a sucrose reward) artificial flowers, in which they needed to learn to ignore unrewarding blue flowers, their innately preferred choice^30^: and to associate yellow as a predictor of food reward. To assess this, we recorded the choices made during 100 sequential flower visits for each bee (see Section S1 for details). The learning assessments usually took place within a few days of a bee beginning to forage on the artificial flowers.

### Free-foraging performance

Each colony was monitored by two observers for three hours a day, five days per week for a total of 20 days. The observation periods were between 0900–1200, 1200–1500 and 1500–1800. The daily order in which colonies were observed was randomised in order to account for differences in forager activity levels over the course of a day. Observation periods consisted of recording the mass of all RFID tagged bees as they walked over a weighing station balance (Fig. S4) when they left or entered the colony. In addition to weighing bees, we recorded the size of any pollen load brought back to the colony; pollen loads were classified as small, medium, large, or very large relative to the size of the bee. We chose this approach because previous trials to remove and weigh pollen loads have disrupted normal foraging activity and worker motivation. Nectar and pollen collection are non-independent due to the physical limitations of carrying both resources^67^, therefore the amount of nectar collected by bees carrying medium to very large pollen loads will be marginal. Using a stopwatch synchronised with the time on the RFID readers, the exact time of each observation was recorded and enabled us to identify individuals^29^.

### Analysis

#### Learning scores

Learning curves, first-order decay functions (y = y0 + Ae^−x/t^), were fitted to flower choice data for each forager (except for one individual that performed particularly poorly, making curve fitting impossible) using Microcal Origin pro 8.6. In this equation ‘x’ is the number of flower choices the bee made after it first feeds from (probes) a yellow flower, and ‘y’ is the number of errors (blue flowers chosen). ‘y0’ is the saturation performance level - the number of errors made by the bee when they reach a performance plateau. ‘t’ is the decay constant of the curve - a measure of learning speed (rate of change in task performance) and ‘A’ is the curve amplitude. A single Learning Performance Index (LPI) was also generated, where we summed the number of errors made (predicted by the learning curve) by each bee when it had made 5, 50 and 100 choices (see Section S2 for details). This produced a LPI score between 0 and 30 errors. Low LPI values are indicative of faster learning while high values indicate slower learners^32^. Our Learning Performance Index is strongly correlated (Spearman’s ρ = 0.619, n = 48, P < 0.001) with ‘t’ (used in previous publications as measure of learning speed^19^), but has the advantage of giving similar weight to the rate of change in performance (slope of the learning curve), the shape of the learning curve, and variation in the level of saturation performance. For this reason we use LPI as our primary measure of learning, but provide model outputs for the following parameters: ‘y0’, ‘t’, and ‘A’ in the Supplementary information (Tables S1–2).

#### Foraging efficiency

Nectar and pollen collection rates were estimated for bees observed to perform at least three nectar- or pollen-collecting bouts (see Section S3 for details on foraging bouts). A nectar collection rate was calculated for each bee returning to the colony without pollen by averaging all incoming weights and subtracting the bee’s average outgoing weight, then dividing this mass difference by the mean trip duration. From weighing pollen foragers it is not possible to determine how much of their forage was pollen and how much was nectar. To generate a measure of pollen collection over time we assigned a numerical value to each of our pollen classifications (i.e. small pollen load = 1, medium = 2, large = 3, very large = 4) and calculated an average pollen load size for each bee across all bouts when pollen was collected. The average pollen load size was then divided by mean trip duration^29^. All statistical analyses were conducted in R v3.0.2 ^68^. Using general linear mixed models GLMMs lme function from the nlme package^69^, we determined whether learning ability predicted *(i) nectar collection rate* and/or *(ii) pollen collection rate*. We adopted a bottom-up model building approach, which is both more conservative than a stepwise deletion approach, and more appropriate given our limited sample size because it avoids over parameterization. Our basic model contained only colony membership as a random effect. This was compared with four different candidate models that contained the basic model and one of the following covariates as a fixed effect: colony age, worker age, worker mass or experience (See Section S4, for definitions). We calculated the AICc value (Akaike Information Criterion – corrected version for small sample sizes) for each model selMod function from the pgirmess package^70^, and the best model of this subset was identified as the model with the lowest AICc value. We then added learning ability to the best model to identify the resultant effect on AICc value. If the AICc was significantly lowered (i.e. ΔAICc >2) by including learning ability we concluded that it was predicting that response variable.

#### Foraging activity

Using the RFID data we determined how frequently each of our tested individuals foraged. Linear mixed models were then used to assess whether learning ability influenced the foraging activity of workers, the response variables included the *(i) mean number of bouts per day*, *(ii) mean bout duration* and *(iii) number of days spent foraging*. The *(i) mean number of bouts* and *(ii) mean bout duration* were square-root transformed (as this improved the model fit) and analysed with a general linear mixed model as described above. A GLMM, with an assumed Poisson error distribution glmer function in lme4 package^71^, was used to analyse count data for *(iii) number of days spent foraging*. Again a basic model was generated and then compared with three additional models that also contained colony age, worker age or worker mass respectively. The residual value of adding learning ability to the previously best model was assessed using AICc scores, and the fit of the best model was confirmed by plotting the fitted vs residual values.

## Electronic supplementary material

Fast learning in free-foraging bumble bees is negatively correlated with lifetime resource collection.
