# Supplementary material for: Fast learning in free-foraging bumble bees is negatively correlated with lifetime resource collection
Source: Sci Rep. 2017 Mar 29;7:496. doi: 10.1038/s41598-017-00389-0 (PMC5428240; doi:10.1038/s41598-017-00389-0)
Supplement: Supplementary file 1 — Fast learning in free-foraging bumble bees is negatively correlated with lifetime resource collection. [file 41598_2017_389_MOESM1_ESM.pdf]

Title: Fast learning in free-foraging bumble bees is negatively correlated with lifetime resource collection.

Supplementary Information

Lisa J. Evans[1,2†*], Karen E. Smith[1†], Nigel E. Raine[1,3]

[1]School of Biological Sciences, Royal Holloway University of London, Egham, Surrey, TW20 0EX, UK

[2]Plant and Food Research, Hamilton, 3240, New Zealand

[3]School of Environmental Sciences, University of Guelph, Guelph, Ontario, N1G 2W1, Canada

† These authors contributed equally to the work.

* Author for correspondence:

lisa.evans@plantandfood.co.nz

Section S1: Visual learning task methods

Bees were pre-trained to forage on 30 bicoloured yellow (Perspex Yellow 260) and blue (Perspex Blue 727) artificial flowers placed in a flight arena (120 x 100 x 35 cm). Each flower (24 x 24 mm: half yellow and half blue) was placed on a glass cylinder (40mm high) to raise them above the floor of the flight arena. During pre-training these flowers were rewarded with 5µl of 50% sucrose solution placed in the middle of the flower. All bees were allowed to forage freely on the flowers and the rewards were replenished as they were consumed, this gave them equal opportunity associate both blue and yellow with reward. Once an individual bee completed at least two consecutive foraging bouts, the bee was let into the arena alone to complete a further three foraging bouts. The number of flowers visited by the bee in each of these bouts was counted and the mean number used to estimate the volume of sucrose solution collected.

Bees were then trained individually in the flight arena with 10 blue and 10 yellow flowers (n = 20 flowers in total). Yellow flowers contained a (50% sucrose solution) reward, while blue flowers were unrewarding (empty). The volume of sucrose solution reward in the yellow flowers was calculated from the mean volume the test bee consumed during the three foraging bouts at the end of the pre-training period. For example, if a bee emptied 20 of the 30 pre-training flowers (20 x 5µl = 100µl), then this volume would be divided equally among the 10 rewarding flowers (10µl/ yellow flower). Bees were regarded as choosing a flower when they either approached or landed on them. Once a bee probed a yellow (rewarding) flower a further 99 choices were recorded, so each bee made sequence of at least 100 flower choices. Visits to (unrewarding) blue flowers were considered as errors, and visits to yellow flowers as correct choices. The choice sequence was captured using EthoLog 2.2.5 software [1] allowing the timing of each choice, and the duration of each foraging bout to be recorded. Flowers were changed between foraging bouts and their positions re-randomised within the flight

arena so that bees were unable to use previous odour cues (scent marks) or spatial position as predictors of reward [2].

Section S2: Calculating learning scores

Learning curves; first-order exponential decay functions ($y = y0 + Ae^{-x/t}$), were fitted to the flower choice data for each bee using Microcal Origin pro 8.6. In this equation ‘x’ is the number of flower choices the bee made after its first yellow flower probe, and ‘y’ is the number of errors. ‘y0’ is the saturation performance level - the number of errors made by the bee when they reach a performance plateau. ‘t’ is the decay constant of the curve - a measure of learning speed (rate of change in task performance) and ‘A’ is the curve amplitude. The starting point for each bee’s learning curve was the proportion of errors made (number of blue flowers chosen) before a bee probed a rewarding yellow flower for the first time. Flower choices made by each bee after and including the first time it probed a rewarding yellow flower were evaluated as number of errors (blue flower choices) in each group of 10 choices. The learning curve was fitted to these 11 data points, i.e. start point and subsequent 10 groups of 10 flower choices, for each individual bee [3]. To generate a single Learning Performance Index (LPI) that took into account the rate of change in performance (slope of the learning curve), the shape of the learning curve and variation in the saturation performance level, we summed the number of errors made by each bee when it had made 5, 50, and 100 choices after probing a rewarding flower for the first time. This produced a learning score out of 30. Low LPI values are indicative of faster learning while high values indicate slower learners [4]. LPI was used as our measure of learning for all analysis, but we also ran the same models with the following parameters: ‘t’, ‘y0’ and ‘A’ (Table S1 and S2 below) for comparison. Results were similar for LPI and ‘t’ (used in previous publications as measure of learning speed: [3]).

Table S2: Candidate models to predict the number of days foraged, mean number of bouts per day and mean bout duration.

	No. of days foraged		Mean bouts per day		Men bout duration	
	AICc	Δ AICc	AICc	Δ AICc	AICc	Δ AICc
Basic	90.59*	12.35	326.25	25.54	161.48	7.80
Worker age	91.14	12.89	322.75	22.05	155.86	2.17
Worker size	93.36	15.12	326.37	25.66	163.53	9.84
Colony age	92.54	14.30	**300.71***	**0**	**153.69***	**0**
Best model + t	**85.20**	**0**	303.06	2.35	155.69	2.35
Best model + y0	91.88	6.68	303.19	2.48	155.63	1.94
Best model + A	92.85	7.65	301.98	1.27	156.15	2.46

The basic model contained only the intercept and colony membership as a random factor. All other models contained the basic model and the additional fixed factors (predictors) specified in the model name. The model with the lowest AICc value out of the five initial models (indicated with an asterisk) had either ‘t’, ‘y0’ or ‘A’ added to it to determine whether this significantly decreased the AICc value (i.e. Δ AICc >2). The best model (based on the AICc value) is shown in bold.

Table S2: Candidate models to predict pollen and nectar collection efficiency by tested bees.

	Mean pollen collection		Men nectar collection	
	AICc	Δ AICc	AICc	Δ AICc
Basic	**-164.33***	**0**	76.77	14.46
Worker age	-162.59	1.74	78.13	15.82
Worker size	-161.82	2.51	79.71	17.41
Colony age	-161.96	2.36	65.90	3.60
Experience	-162.54	1.79	**62.31***	**0**
Best model + t	-157.41	6.92	65.59	3.28
Best model + y0	-157.60	6.73	63.65	1.34
Best model + A	-158.17	6.16	62.48	0.17

The basic model contained only the intercept and colony membership as a random factor. All other models contained the basic model and the additional fixed factors (predictors) specified in the model name. The model with the lowest AICc value out of the five initial models (indicated with an asterisk) had either ‘t’, ‘y0’ or ‘A’ added to it to determine whether this significantly decreased the AICc value (i.e. Δ AICc >2). The best model (based on the AICc value) is shown in bold.

Section S3: Assessing foraging efficiency and activity

Using data from the Radio Frequency Identification (RFID) system we calculated the mean number of foraging bouts completed per day by each of our tested individuals. As only foragers were RFID-tagged assumed that all trips away from the nest were foraging bouts, provided that the bee was gone for ≥ 8min and upon their return they remained in the nest for ≥ 1 min. (to off-load pollen and/or nectar). Whilst some previous authors have used 10 minutes as their foraging bout threshold[5,6] we chose eight minutes because some bees (n = 7) consistently made foraging trips lasting around eight minutes. To explore whether this bout threshold level would affect our results (by potentially including non-foraging trips) we repeated the analysis using only bouts ≥ 10 min and found no change to the relationships reported. If a forager entered a second colony ≤ 5 min after exiting a colony, it was assumed that this was its correct colony and the previously visited colony was an orientation error. In these instances the foraging bout concluded at the second colony. When observing colonies

this appeared to be the case because foragers (which we could often identify using their coloured tags underneath the RFID tags) would enter the second colony still carrying their pollen or would have similar entry weights, which suggests they had not off-loaded nectar (or pollen) in the first colony. An exception to this rule was if a forager spent more time in the first colony it entered. When this occurred it was assumed that the visit to the second colony was the orientation error.

Foragers were observed to move between colonies during their foraging career. Such 'drifting' is a common occurrence between closely situated colonies [7] and comparable levels of drifting were observed by Gill et al. [8] in an experiment using a similar experimental setup. To take drifting into account, we identified the colony each forager visited for the majority of its foraging trips (the majority colony). On average, foraging bees performed 61.88% ± 2.33 of their foraging bouts for their majority colony, compared to 37.36% ± 3.51 for their natal colony. Based on these results we felt that majority colony was a more accurate indicator of colony membership and used this measure in our models.

Section S4: Fixed effects in GLMMs

Colony age the number of days since the colony arrived in the lab when each focal worker was assessed and RFID tagged.

Worker size was obtained by averaging all outgoing mass recordings for each forager. Bees for which we had no observation data for (usually bees that completed very few foraging bouts) were assigned an outgoing body mass based on the average bee mass for their natal colony (n = 4).

Worker age was either known, if they were tagged on the day of emergence, or estimated, if they were present when the colony arrived (n = 10). Estimated ages were calculated by adding five days to the colony arrival date.

Experience was calculated for each bee by averaging the number of previous foraging trips made prior to the foraging bouts we observed. For example, if we observed a bee's 5th, 22nd, 35th, and 40th bouts, these were averaged to give an experience score of 25.5.

Figure S1. The divided colony box has four chambers and a mesh partition (2) that separates the two halves of the same colony. There are two chambers for each half of the colony, the inner chamber is their 'nesting' area (1 – containing the brood and food stores) the other was used as a temporary feeding chamber (3). One half of the colony was connected to a laboratory flight arena (internal side). Once the learning speed of each forager had been assessed, the bee was RFID tagged and moved to the other (external) side of the mesh partition. Bees on this side of the nest could leave to forage outside the laboratory. Each chamber was covered with transparent Perspex to enable observations. When not in use the chambers were also covered with a square of cardboard to create a dark environment similar to the natural nesting condition of subterranean bumble bees. When transferring tested bees from the internal side of the colony the chance of aggression was minimised by transferring them into the second chamber (3) on the external side of the divided colony box (rather than placing directly amongst the bees and brood). The two chambers were connected (3 & 1), allowing the transferred bee to integrate itself with colony members.

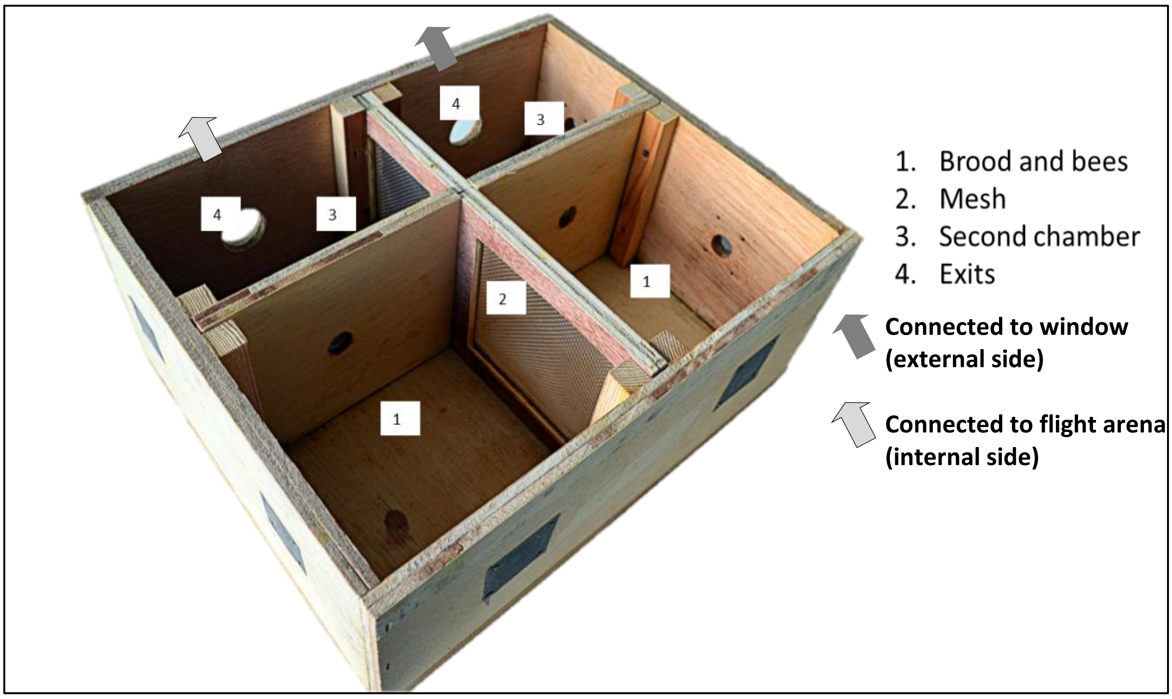
4
3
4
3
2
1
1
1. Brood and bees
2. Mesh
3. Second chamber
4. Exits
Connected to window (external side)
Connected to flight arena (internal side)

Figure S2. Experimental setup. The internal side (1) of the divided colony box is connected to a flight arena and the external side (2) of the colony box was connected to a window via a tunnel made up of several different components. A length of clear, flexible, tubing (3) was connected to the front of the colony box and then to a clear Perspex tunnel (24 x 3 x 3 cm), which was positioned just above a weighing station (5: see also Figure S4 for details). After passing through the weighing station an outgoing forager would pass through two Radio-Frequency Identification (RFID) readers (4). Using the two readers we could identify the direction of a tagged forager's movement (i.e. leaving or entering the colony) and record the exact time and RFID number of the tagged workers passing underneath [8,9]. Lastly, a ridged (ribbed) outlet tube (8) ran from the weighing station up to the window (9). Outside the window was a landing platform (below the exit hole). Above the exit hole was a unique black and white pattern (chosen to minimise any bias in colour return rates based on innately preferred colours: Figure S3) to assist returning foragers to orientate back to their nest. The minimum distance between exits was 38cm.

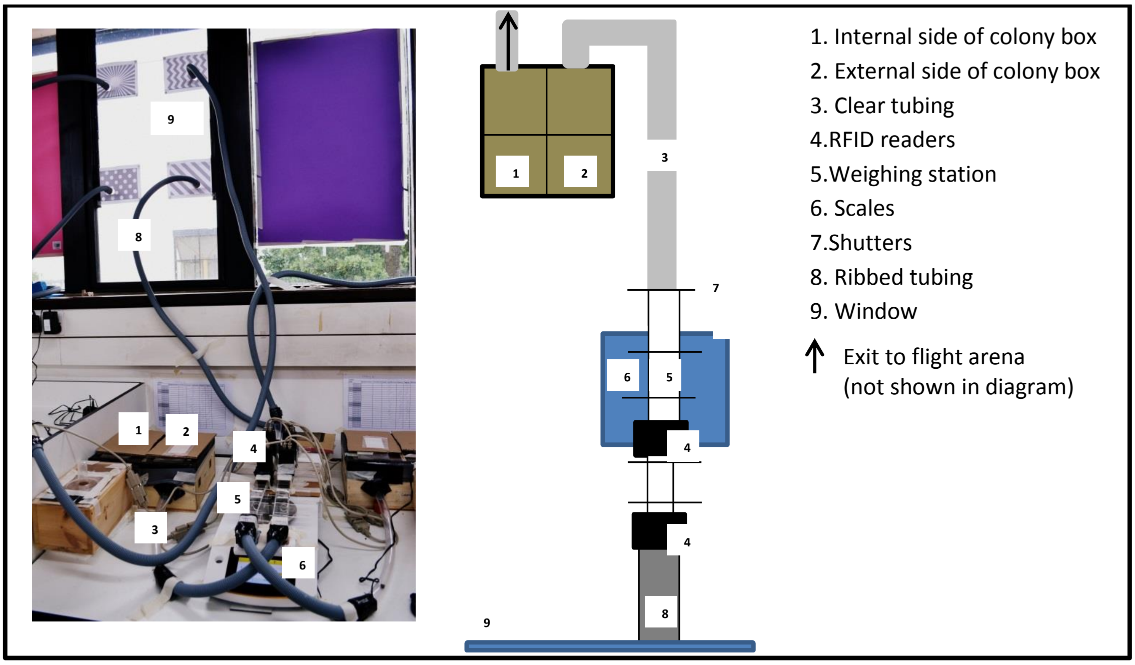

Figure S3. View of an exit hole with a landing platform and its unique black and white pattern from outside the laboratory window.

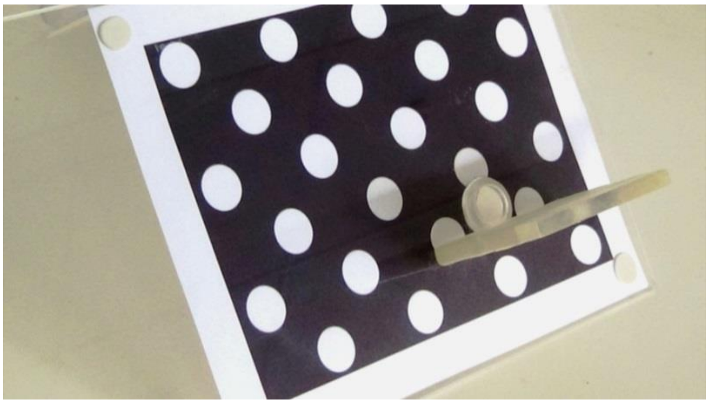

Figure S4. Weighing station setup. Each weighing station contained a number of shutters (one indicated with an arrow) to enable foragers traffic to be controlled and temporarily restrict individual foragers above the balance. As a forager walked over the balance pan it could be weighed using the dynamic weighing function (designed for weighing moving animals). Three mass recordings were taken for each bee and the average of these values used in the analysis.

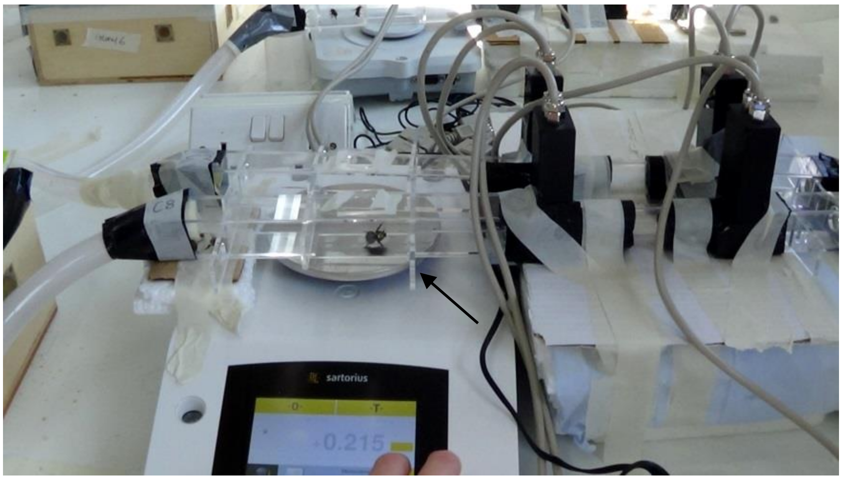

Figure S5. Box and whisker plots showing foraging bout duration at different stages during in the bee's foraging career, beginning with their first foraging bout and ending with their last. The dataset includes all bees that completed more than 15 bouts (n = 46) to increase the accuracy of estimating 'proportion of total foraging bouts' (in effect this excluded only three foragers). The bottom and top of each blue box indicates the 25th and 75th percentile (IQR), the thick line horizontal indicates the median, and the whiskers indicate minimum and maximum values. The "circles" at the end of the boxplot represent outliers (±1.5*IQR).

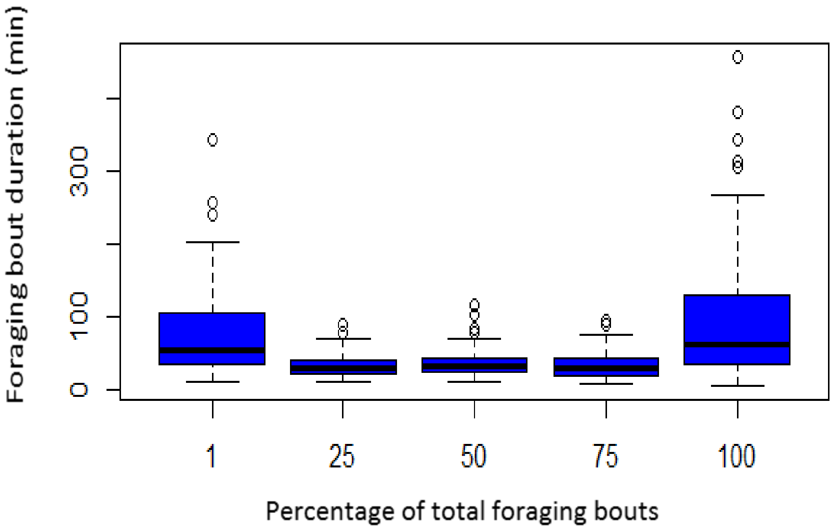

Figure S6. Average daily humidity % (dashed line), air temperature °C (black line), and rainfall mm (grey line) over the duration of our observation period.

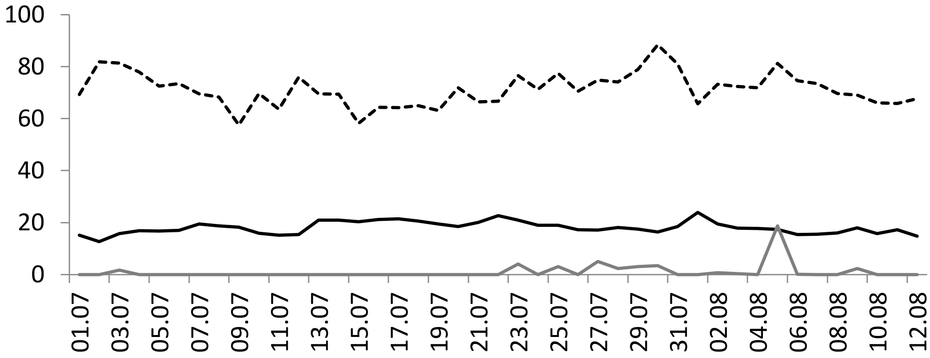

References

1 Ottoni, E. B. EthoLog 2.2: A tool for the transcription and timing of behavior observation sessions. *Behavior Research Methods Instruments & Computers* **32**, 446-449, doi:10.3758/bf03200814 (2000).

2 Saleh, N. & Chittka, L. The importance of experience in the interpretation of conspecific chemical signals. *Behavioral Ecology and Sociobiology* **61**, 215-220, doi:10.1007/s00265-006-0252-7 (2006).

3 Raine, N. E. & Chittka, L. The correlation of learning speed and natural foraging success in bumble-bees. *Proceedings of the Royal Society B* **275**, 803-808, doi:10.1098/rspb.2007.1652 (2008).

4 Evans, L. J. & Raine, N. E. Changes in learning and foraging behaviour within developing bumble bee (*Bombus terrestris*) colonies. *PLoS One* **9**, doi:10.1371/journal.pone.0090556 (2014).

5 Capaldi, E. A. & Dyer, F. C. The role of orientation flights on homing performance in honeybees. *Journal of Experimental Biology* **202**, 1655-1666 (1999).

6 Peat, J. & Goulson, D. Effects of experience and weather on foraging rate and pollen versus nectar collection in the bumblebee, *Bombus terrestris*. *Behavioral Ecology and Sociobiology* **58**, 152-156, doi:10.1007/s00265-005-0916-8 (2005).

7 Zanette, L. R. S., Miller, S. D. L., Faria, C. M. A., Lopez-Vaamonde, C. & Bourke, A. F. G. Bumble bee workers drift to conspecific nests at field scales. *Ecological Entomology* **39**, 347-354, doi:10.1111/een.12109 (2014).

8 Gill, R. J., Ramos-Rodriguez, O. & Raine, N. E. Combined pesticide exposure severely affects individual and colony level traits in bees. *Nature* **491**, 105-108, doi:10.1038/nature11585 (2012).

9 Molet, M., Chittka, L., Stelzer, R. J., Streit, S. & Raine, N. E. Colony nutritional status modulates worker responses to foraging recruitment pheromone in the bumblebee *Bombus terrestris*. *Behavioral Ecology and Sociobiology* **62**, 1919-1926, doi:10.1007/s00265-008-0623-3 (2008).
